# Dexmedetomidine and intravenous acetaminophen for the prevention of postoperative delirium following cardiac surgery (DEXACET trial): protocol for a prospective randomized controlled trial

**DOI:** 10.1186/s13063-018-2718-0

**Published:** 2018-06-22

**Authors:** Puja Shankar, Ariel Mueller, Senthil Packiasabapathy, Doris Gasangwa, Melissa Patxot, Brian O’Gara, Shahzad Shaefi, Edward R. Marcantonio, Balachundhar Subramaniam

**Affiliations:** 1 Center for Anesthesia Research Excellence (CARE), Department of Anesthesia, Critical Care and Pain Medicine, Beth Israel Deaconess Medical Center, One Deaconess Road, CC-650, Boston, MA 02215 USA; 2 Harvard Medical School, 25 Shattuck Street, Boston, MA 02215 USA; 3 Division of General Medicine and Primary Care, Beth Israel Deaconess Medical Center, Harvard Medical School, 330 Brookline Avenue, Boston, MA 02215 USA

**Keywords:** Postoperative delirium, Neurocognition, Cardiac surgery, Coronary artery bypass grafting, Montreal Cognitive Assessment, Confusion Assessment Method, Propofol, Acetaminophen, Dexmedetomidine

## Abstract

**Background:**

Postoperative delirium is common in elderly cardiac surgery patients. It is multifactorial and is influenced by the patient’s baseline status and the nature of the medical and surgical interventions that the patient receives. Some of these factors are potentially modifiable, including postoperative sedation and analgesia protocols. This study has been designed to evaluate the effectiveness of postoperative intravenous acetaminophen in conjunction with either dexmedetomidine or propofol in decreasing the incidence of delirium.

**Methods:**

This is a prospective, randomized, placebo-controlled, double-blinded, factorial trial that includes patients who are at least 60 years old and who are undergoing cardiac surgeries involving cardiopulmonary bypass, including coronary artery bypass graft (CABG) and combined CABG/valve surgeries. Patients are randomly assigned to receive one of four postoperative analgesic-sedation regimens: (1) acetaminophen and dexmedetomidine, (2) acetaminophen and propofol, (3) dexmedetomidine and placebo, or (4) propofol and placebo. The primary outcome, incidence of delirium, will be assessed with the Confusion Assessment Method (CAM or CAM-ICU). The secondary outcome, postoperative cognitive decline, will be assessed with the Montreal Cognitive Assessment. Additional secondary outcomes, including duration of delirium, postoperative analgesic requirement, length of stay, and incidence of adverse events, will also be reported. Data will be analyzed in 120 randomly assigned patients who received at least one dose of the study medication(s) on a modified intention-to-treat basis.

**Discussion:**

This study has been approved by the institutional review board at Beth Israel Deaconess Medical Center, and the trial is currently recruiting. This study will systematically examine the implications of modification in postoperative sedative/analgesic protocols after cardiac surgery, specifically for short- and long-term cognitive outcomes. Any positive outcomes from this study could direct simple yet effective practice changes aimed to reduce morbidity.

**Trial registration:**

ClinicalTrials.gov Identifier: NCT02546765, registered January 13, 2015.

**Electronic supplementary material:**

The online version of this article (10.1186/s13063-018-2718-0) contains supplementary material, which is available to authorized users.

## Background

Delirium is defined as a change in mental status, characterized by acute onset and fluctuating course, inattention, disorganized thinking, and altered level of consciousness [1]. The incidence of delirium varies with the setting and the population analyzed and is reported to be as high as 82% in the intensive care unit (ICU) [2]. Compounding this, the incidence ranges from 11% to 46% among all cardiac surgical patients and is even higher in the geriatric population undergoing cardiac surgery. In-hospital delirium confers a significant morbid burden and has been correlated with poor hospital outcomes, including significant increases in mortality [2], nosocomial complications, and longer ICU and hospital stays [3, 4]. Delirium has also been associated with debilitating long-term outcomes, including poor functional recovery, and accelerated cognitive decline with impaired cognitive function for up to 1 year after cardiac surgery [5].

Risk factors for delirium in patients undergoing cardiac surgery include pre-existing cognitive dysfunction, depression, transient ischemic attacks, and abnormal serum albumin [6]. The interplay between these pre-existing comorbid conditions and numerous perioperative factors, including postoperative pain, mechanical ventilation, use of deliriogenic analgesic and sedative drugs, and the inflammatory response to surgery and cardiopulmonary bypass, makes the elderly especially vulnerable to delirium. Despite this, delirium is deemed preventable in 30% to 40% of cases [2]. Although the pathogenesis of postoperative delirium is not completely understood, modifiable factors such as perioperative sedation and opioid-based analgesia may contribute to the etiology [7, 8].

Traditionally used sedatives and analgesics, namely benzodiazepines and opioids, have psychoactive properties. Midazolam, a benzodiazepine, acts via gamma-aminobutyric acid (GABA) receptors and is found to release deliriogenic mediators [9]. Additionally, opioids such as morphine have long been known to cause delirium [8]. However, dexmedetomidine, a selective alpha-2 receptor agonist, provides analgesia and sedation without causing respiratory depression and has no effect on the GABA receptors. Dexmedetomidine is also suggested to have an opioid-sparing effect [10] and anti-inflammatory properties [11]. Studies have also demonstrated reduction in mortality with the use of dexmedetomidine in cardiac surgery [12]. Although these characteristics suggest dexmedetomidine to be a unique and promising drug for sedation following cardiac surgery, the studies investigating its effect on the incidence and duration of delirium have thus far shown mixed results. Non-standardized [13] and insensitive outcome measurements in non-intubated patients [14] have contributed to the mixed results. A recent meta-analysis suggested that the use of dexmedetomidine for sedation in cardiac surgery patients may reduce postoperative delirium [9]. However, many of the studies included did not have a robust design and used various outcome measures and tools to assess delirium. Another study found no significant decrease in the incidence of postoperative delirium with the use of dexmedetomidine but was likely underpowered [15]. A recent study compared postoperative sedation using dexmedetomidine versus propofol and the associated delirium incidence. They found lower incidence of delirium in the dexmedetomidine group compared with the propofol group [16]. There are more studies that examine the role of dexmedetomidine in the incidence of postoperative delirium, but the results are contradicting. In a large trial that studied the use of intraoperative dexmedetomidine infusion, which was continued for 2 h into the postoperative period, in non-cardiac surgical patients, no difference was found in the incidence of postoperative delirium compared with the saline placebo [17]. The authors have emphasized the importance of the timing of dexmedetomidine infusion. Another large trial compared the effect of dexmedetomidine infusion in the postoperative period to placebo. They found a significant decrease in delirium in the first seven postoperative days in the dexmedetomidine group [18]. Hence, there is no clear consensus on the usefulness of dexmedetomidine in delirium prevention.

Postoperative pain following cardiac surgery is attributable to many factors, including the skin or sternal incision, sternal retraction, pericardial incision, chest tube sites, and leg incisions for graft harvesting [19]. The current pain relief strategy primarily includes postoperative opioid administration (morphine or hydromorphone), although a multimodal balanced postoperative pain management strategy could prove advantageous. Although inadequate pain relief can increase the risk of postoperative delirium [20], central nervous system properties of opioids can also increase this risk, especially in elderly patients [21]. Non-steroidal anti-inflammatory drugs (NSAIDs), including acetaminophen, are the other agents used in a multimodal regimen. NSAIDs are generally avoided after cardiac surgery because of their deleterious effects on the gastric mucosa and kidney and increased risk of bleeding. Intravenous (IV) acetaminophen is a good alternative, and no bleeding risk is associated with its use. It inhibits cyclo-oxygenase and consequently the synthesis of pro-inflammatory cytokines [22]. It also decreases production of inflammatory agents such as histamine and leukotrienes [23]. In addition to its peripheral effects, it may also have effects on the central nervous system which contribute to its analgesic property [24]. Billings et al. previously showed that perioperative administration of acetaminophen decreases lipid peroxidation during cardiopulmonary bypass [25]. This study explores the effect of cardiopulmonary bypass and its inflammatory response leading to erythrocyte lysis and the effect of acetaminophen on its prevention [25]. Although the context is different, the attenuation of lipid peroxidation with secondary inflammation and injury in the immediate postoperative period as seen with acetaminophen may be relevant to its effects on the central nervous system and subsequently on the incidence of delirium. The use of IV acetaminophen has been shown to decrease opioid consumption after surgeries such as hip and knee replacements, cesarean sections, and coronary artery bypass surgeries [23, 26]. Despite these promising findings, IV acetaminophen has never been studied in the context of delirium prevention following cardiac surgery.

This study aims to assess the role of postoperative pharmacological interventions in decreasing the incidence of delirium. We hypothesize that the use of postoperative acetaminophen and dexmedetomidine will result in a decrease in the incidence of postoperative delirium after cardiac surgery.

## Methods

### Study design

This article includes all components as described in the SPIRIT (Additional file 1) checklist [27]. This is a prospective, randomized, placebo-controlled, double-blinded, factorial, exploratory trial to study the effect of IV acetaminophen and dexmedetomidine (compared with the standard analgesic/sedation regimen of opioids and propofol) on the incidence of postoperative delirium and cognitive decline following cardiac surgery. Subjects undergoing coronary artery bypass grafting (CABG) or combined CABG and valve (aortic or mitral or both) procedures will be recruited after written informed consent is obtained. Informed consent for participation and for blood collection will be obtained by a trained study staff member.

**Fig. 1:**
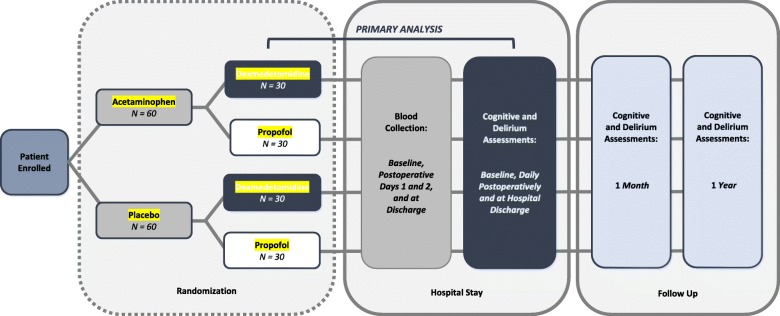
Study schema

**Fig. 2:**
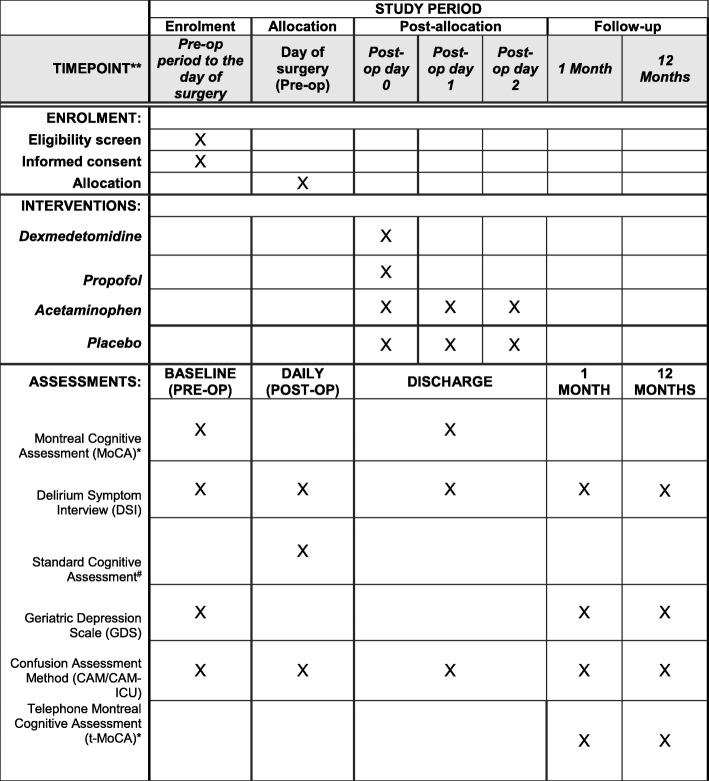
Standard Protocol Items: Recommendations for Interventional Trials (SPIRIT) figure: The schedule of enrollment, interventions, and assessments in the study

The primary outcome, incidence of postoperative delirium, will be determined by assessing patients at baseline, daily postoperatively, and on the day of discharge. Incidence of postoperative cognitive decline will also be measured with neurocognitive assessments at similar intervals. In addition, a follow-up neurocognitive assessment will be offered at 1 month and 1 year post-discharge by phone. The study schema is represented in Fig. 1. The SPIRIT figure (Fig. 2) demonstrates the schedules of enrollment, interventions, and assessments used in the trial.

### Study registration

This study protocol was approved by the Committee on Clinical Investigations Institutional Review Board (IRB) at Beth Israel Deaconess Medical Center (Protocol 2014-P-000413). This trial was registered with the US National Institutes of Health on ClinicalTrials.gov with the trial identification number NCT02546765 on January 13, 2015. The trial is ongoing and recruiting.

### Inclusion and exclusion criteria

Patients who are at least 60 years old and who are undergoing cardiac procedures, namely CABG with or without valve replacement (aortic or mitral or both), that require bypass will be included in the study. Patients with any of the criteria listed in Table 1 will be excluded.

**Table 1:** Study exclusion criteria

Preoperative left ventricular ejection fraction less than 30%	
Emergent procedure	
Isolated aortic surgery	
Pre-existing cognitive impairment	
Parkinson’s disease	
Alzheimer’s disease	
Recent seizures (<3 months)	
Prophylactic medications for cognitive decline	
Serum creatinine greater than 2 mg/dL	
Liver dysfunction (liver enzymes more than four times the baseline or history and exam suggestive of jaundice)	
Active (in the past year) history of alcohol or drug abuse (>10 drinks per week)	
Hypersensitivity to any of the study medications	
Non–English-speaking (inability to complete the cognitive assessments)	

### Randomization and study intervention

Blocked randomization will be used to assign recruited participants to one of four combinations of analgesics and sedatives in a 1:1:1:1 allocation: (1) acetaminophen and dexmedetomidine, (2) acetaminophen and propofol, (3) placebo and dexmedetomidine, or (4) placebo and propofol (Fig. 1). Randomization is accomplished by using a sequence of computer-generated random numbers, which is used by the research pharmacy to assign the participant to a group.

All preoperative and intraoperative management will be at the discretion of the treating provider and will adhere to the current institutional standard of care for cardiac surgery. All study medications will be administered intravenously, and doses will be weight-based. Patients in either of the dexmedetomidine groups will get a bolus dose of 0.5 to 1 μg/kg during chest closure which will be followed by a maintenance infusion of 0.1–1.4 μg/kg per h into the postoperative period. Propofol infusions will be avoided in these patients. Patients in either of the propofol groups will receive propofol at a dose of 20–100 μg/kg per min for postoperative sedation. Both the sedatives will be started during chest closure and continued for up to 6 h postoperatively or until the extubation of the patient, whichever occurs earlier. IV acetaminophen at a dose of 1 g or placebo of similar volume will be administered immediately after the patient is transferred to the cardiovascular ICU and will be repeated every 6 h until the first 48 h for a total of eight doses. Both acetaminophen and placebo (0.9% saline) will be distributed in an IV bag in equal volumes, thus effectively blinding the nurses and researchers to the administration of acetaminophen. Owing to the easily recognizable nature of the propofol emulsion, there will be no blinding to the sedative administered (propofol or dexmedetomidine). Study drug administration and adherence to group assignment will be monitored and recorded by study staff periodically.

### Study outcomes and their measures

#### Primary endpoint

The primary outcome for this study is the incidence of delirium during the patients’ postoperative hospital stay. Incidence of delirium will be assessed by using Confusion Assessment Method (CAM). CAM-ICU will be used for intubated patients. Once consent is obtained, a baseline delirium assessment will be administered with days of the week backwards (DOWB) and months of the year backwards (MOYB), the Delirium Symptom Interview (DSI), the Geriatric Depression Scale (GDS), and the CAM. At the same time, the Montreal Cognitive Assessment (MoCA) will be completed in order to assess cognitive function as a secondary outcome. With the exception of the GDS, the same assessments will be administered at discharge.

Postoperatively, the patient will complete a standard cognitive assessment, which will include a CAM (or CAM-ICU) and DSI. Delirium assessment will be performed once every day starting from postoperative day 1. If the CAM assessments reveal absence of delirium for three consecutive days, the subject will fall into a skip pattern and only complete the assessment every other day to reduce examination fatigue. Additionally, a follow-up assessment will be administered at 1 month and 1 year post-discharge and will include a telephone version of the MoCA (t-MoCA), DSI, GDS, and CAM. In order to limit loss to follow-up for our secondary outcomes, the protocol permits a window period of 14 days before and after the 1-month follow-up and of 3 months before and after the 1-year follow-up. Assessments will be administered and scored by blinded study team members who are extensively trained in the administration of neurocognitive and delirium assessments. Study team members administering cognitive assessments as well as those monitoring safety will be blinded as to whether or not the patient received acetaminophen or placebo. The time points of various assessments are presented in Fig. 2.

#### Secondary endpoints

Secondary outcomes include duration of delirium, postoperative cognitive decline (assessed by using MoCA scores), breakthrough analgesic requirements within the first 48 h postoperatively, and ICU/hospital length of stay. The potential adverse effects of the study drugs, such as the number and duration of episodes of hypotension (predefined as less than 90 mm Hg systolic blood pressure for 5 min or more) and bradycardia (predefined as less than 40 beats per minute), will be collected from the patient’s medical records. The proportion of patients who complete the protocol successfully will be measured. If this sedation and analgesic regimen is abandoned by the clinicians, the reason will be qualitatively described at the end of the study.

#### Blood collection and processing

Blood samples will be collected from all participants at four time points, namely at baseline, at postoperative days 1 and 2, and at the time of hospital discharge. Baseline blood samples will be collected at the time of enrollment or on the day of surgery before anesthetic drug administration. Discharge blood samples will be collected within 48 h prior to discharge or postoperative day 8 to 10, whichever comes first. A total volume of 20 cm^3^ of blood will be obtained in two purple-top ethylenediaminetetraacetic acid tubes. Samples will be centrifuged, and the plasma and buffy coat will be separated from red blood cells, aliquoted into smaller cryovials, labeled, and stored at − 80°C for future analysis of inflammatory biomarkers.

#### Safety monitoring

Patients may be withdrawn or returned to the standard of care at any time if there is a significant clinical indication to do so. Throughout the intraoperative and immediate postoperative period, the study team will be in communication with the clinical team to ensure protocol adherence and safety. Trained research team members will monitor protocol compliance and report adverse events to the IRB. Adverse events will be assessed daily by trained staff members during the hospital stay for a maximum of 10 days. Since the patients in this population are undergoing high-risk surgery and are critically ill, it is expected that they will have several unrelated adverse health events during their stay in the hospital. Consequently, we will limit the scope of our adverse event monitoring and reporting to all serious adverse events, including unexpected death, believed to be related to the study intervention as well as non-serious adverse events believed to be possibly or probably related to the study intervention. In case the patient experiences a study drug–related adverse event, such as a severe allergic reaction, an unblinding protocol will be followed. The patient randomization number will be used to find the drug assignment from a code-breaker and to alert the treating provider regarding the same.

#### Data collection

Demographic data such as age, sex, ethnicity, race, and body mass index will be recorded. Patient comorbid conditions, surgery, and anesthetic drugs will be obtained from the patient’s medical records as well as from the Society of Thoracic Surgery database (https://www.sts.org/registries-research-center/sts-national-database/adult-cardiac-surgery-database/data-collection), which is a clinical outcomes registry that records and assesses the care of adult patients undergoing cardiac procedures at participating hospitals. It includes patient characteristics such as preoperative medications, comorbidities, surgical characteristics, and postoperative outcomes, including 30-day mortality and major adverse events like renal failure during hospital admission. Safety data, including incidence of bradycardia, hypotension, and other adverse events believed to be possibly related to the study intervention, will be recorded. In addition, information on sedatives and analgesics given within 6 h prior to initiation of the cognitive assessment will be recorded in order to determine whether there is an effect on performance. The total opioid dosage administered during first 48 h postoperatively will be recorded. These data, along with pain scores and patient delirium assessment data, will be collected by using Research Electronic Data Capture (REDCap). REDCap is a web-based application that allows customized data collection and entry. The study team will build and maintain the electronic case report form data and ensure data completeness and quality periodically by means of internal audits. Double data entry using a data monitoring arm on REDCap will be used to ensure data quality. All efforts will be made to maintain confidentiality of patient data by using multiple means such as de-identification, use of password-protected secure servers, and restriction of access to study team members.

#### Sample size calculation

Previous studies have shown the incidence of postoperative delirium after cardiac surgeries to vary between 11% and 46% [2]. A pilot study (*N* = 12) was conducted at our institution [28] to compare the effect of acetaminophen and dexmedetomidine administration with that of the standard propofol and opioid regimen on the incidence of delirium. There was a 50% incidence of delirium observed in this pilot study. A 66% reduction in the incidence of delirium with the use of IV acetaminophen was also noticed. In an effort to be slightly more conservative, the current study was powered to detect a 50% reduction in the delirium incidence. With an alpha of 0.05 and 80% power, a sample size of 58 patients will be needed per group of acetaminophen or placebo (with or without dexmedetomidine). Because complete data from 116 patients are required in order to do an analysis, we plan to enroll enough patients to obtain data from 120 patients who receive the study intervention (30 patients in each of the four subgroups). We estimate that about 150 patients will be enrolled (consented) in order to achieve this sample size, as patients can be withdrawn both before randomization (i.e., canceled surgery) and after randomization but before receiving the study intervention (i.e., change in surgery no longer requiring bypass intraoperatively). We will stop enrollment once a total of 120 randomly assigned patients have undergone surgery and received one of the study medications.

#### Data analysis

The primary outcome will be the incidence of postoperative delirium. Our pre-specified primary analysis will assess the incidence of delirium between patients receiving IV acetaminophen versus placebo. All four sedative/analgesic groups will be compared in terms of delirium incidence. Variables will be presented as mean ± standard deviation, median [interquartile range], or frequencies and proportions depending on variable type and distribution. Normality will be assessed with a Shapiro-Wilk test. Dichotomous variables will be assessed by using a chi-squared test. Continuous variables (e.g., length of stay, duration of delirium, age, and analgesic consumption) will be assessed by using parametric *t* tests or the Wilcoxon rank-sum test. The proportion of patients who successfully complete the sedation/analgesic protocol will be analyzed. The reasons for failure to follow the protocol will be qualitatively ascertained from the treating providers. Logistic regression will be employed to assess the relationship of delirium incidence between groups and will be reported as odds ratios and 95% confidence intervals. Given the nature of a randomized controlled trial, we do not anticipate seeing differences between groups in baseline characteristics; however, we will perform a secondary analysis, adjusting for any differences observed between groups and present adjusted odds ratios. SAS 9.4 (SAS Institute Inc., Cary, NC, USA) will be used for all analyses, and *P* values of less than 0.05 will be considered statistically significant. Our primary analysis will be conducted on a modified intention-to-treat basis, in which all patients who received at least one dose of the study drug will be analyzed.

## Discussion

### Significance

This is a large, randomized, controlled, double-blinded study that aims to assess whether there is a relationship between IV acetaminophen and postoperative delirium and whether this relationship is dependent on the sedative used. Study outcomes are measured by using tools that are tested and proven to be sensitive in identifying delirium. They are designed to detect any cognitive changes in the past 24 h and hence are performed on a daily basis. We have also incorporated a cognitive assessment to measure postoperative cognitive decline over the course of the immediate and extended postoperative period. Furthermore, the follow-up at two time points may provide insight into the long-term effects of the study intervention on delirium and neurocognition. If the benefits proposed are substantiated, this can have a significant impact on patient care and satisfaction following cardiac surgery, as it involves modifiable factors that could be easily incorporated into clinical practice.

### Limitations

This trial is designed to study the role of postoperative pharmacological intervention, and any sedative intervention in the preoperative and intraoperative period is left to the discretion of the treating provider. This may have a potential to impact the outcome. As with any study assessing changes over time, there is the potential for loss to follow-up to occur. This limitation is minimized by the fact that it may largely impact our secondary rather than the primary outcome. Furthermore, in an effort to mitigate non-response, we have established wide windows to allow for completion of the follow-up assessments and have also limited the number of follow-up assessments to two. Additionally, we have accounted for dropouts in our sample size calculations. Another potential limitation of this study is the inability to blind the choice of sedative used (propofol or dexmedetomidine) to the study team members. This study includes only one academic medical center and its patient population. While we are located in an urban area, the study population may not be a completely representative sample of those who undergo these types of procedures. Furthermore, clinical care guidelines may differ from those of other hospitals, especially in terms of sedation and pain management. Despite these limitations, this study will provide further insight into the utility of IV acetaminophen with or without dexmedetomidine in reducing the incidence of postoperative delirium following cardiac surgery.

### Ethics and dissemination

The drugs used in the study are US Food and Drug Administration–approved for sedation and analgesia and are routinely used as standard of care in the cardiac postoperative setting. In the tightly monitored environment of the cardiovascular ICU, if any drug-related adverse event occurs, it can be detected early and managed effectively. Thus, it is expected that the benefits of the intervention would far outweigh the anticipated risks associated with them.

Phlebotomy is associated with a small risk of pain and bruising. So whenever possible, blood draws are performed via existing arterial or central venous lines inserted for the purpose of surgery or are obtained at the same time as blood draws for other clinical lab work. As for the volume, a maximum of 80 cm^3^ of blood is drawn over the duration of hospital stay, which is considered minimal risk.

There is chance of patient fatigue with daily cognitive assessments. To overcome this, the patients are placed on an alternate day assessment pattern once they are CAM-negative (delirium-free) for three consecutive days.

This study has been approved by the Committee on Clinical Investigations Institutional Review Board at Beth Israel Deaconess Medical Center (Protocol 2014-P-000413). Written informed consent will be obtained from all subjects prior to initiation of study procedures.

We have planned to present the results of this study in national/international meetings and to publish them in scientific journals. The datasets used or analyzed during the current study will be available from the corresponding author upon reasonable request. There are no plans to individually notify participants regarding the results of this study.

#### Trial status

The trial is ongoing and currently recruiting.

## Additional file

Additional file 1: Standard Protocol Items: Recommendations for Interventional Trials (SPIRIT) checklist. The document represents the SPIRIT Checklist. (DOC 135 kb)

## Abbreviations

CABG: Coronary artery bypass graft
CAM: Confusion Assessment Method
CAM-ICU: Confusion Assessment Method–Intensive Care Unit
DSI: Delirium Symptom Interview
GABA: Gamma-aminobutyric acid
GDS: Geriatric Depression Scale
ICU: Intensive care unit
IRB: Institutional review board
IV: Intravenous
MoCA: Montreal Cognitive Assessment
NSAID: Non-steroidal anti-inflammatory drug
REDCap: Research Electronic Data Capture
SPIRIT: Standard Protocol Items: Recommendations for Interventional Trials
